# Assessing Species Diversity Using Metavirome Data: Methods and Challenges

**DOI:** 10.1016/j.csbj.2017.09.001

**Published:** 2017-09-21

**Authors:** Damayanthi Herath, Duleepa Jayasundara, David Ackland, Isaam Saeed, Sen-Lin Tang, Saman Halgamuge

**Affiliations:** a Department of Mechanical Engineering, University of Melbourne, Parkville, 3010 Melbourne, Australia; b Department of Computer Engineering, University of Peradeniya, Prof. E. O. E. Pereira Mawatha, Peradeniya, 20400, Sri Lanka; c School of Public Health and Community Medicine, University of New South Wales, Randwick, NSW 2052, Australia; d Department of Biomedical Engineering, University of Melbourne, Parkville, 3010 Melbourne, Australia; e Biodiversity Research Center, Academia Sinica, Nan-Kang, Taipei 11529, Taiwan; f Research School of Engineering, College of Engineering and Computer Science, The Australian National University, Canberra 2601, ACT, Australia

**Keywords:** Metagenomics, Phage studies, Biodiversity, Species diversity, Metavirome data, Bioinformatics

## Abstract

Assessing biodiversity is an important step in the study of microbial ecology associated with a given environment. Multiple indices have been used to quantify species diversity, which is a key biodiversity measure. Measuring species diversity of viruses in different environments remains a challenge relative to measuring the diversity of other microbial communities. Metagenomics has played an important role in elucidating viral diversity by conducting metavirome studies; however, metavirome data are of high complexity requiring robust data preprocessing and analysis methods. In this review, existing bioinformatics methods for measuring species diversity using metavirome data are categorised broadly as either sequence similarity-dependent methods or sequence similarity-independent methods. The former includes a comparison of DNA fragments or assemblies generated in the experiment against reference databases for quantifying species diversity, whereas estimates from the latter are independent of the knowledge of existing sequence data. Current methods and tools are discussed in detail, including their applications and limitations. Drawbacks of the state-of-the-art method are demonstrated through results from a simulation. In addition, alternative approaches are proposed to overcome the challenges in estimating species diversity measures using metavirome data.

## 1 Introduction

Most viruses in the environment exist in the form of parasites that infect prokaryotes and hence are frequently termed phages or bacteriophages. Recent studies [1], [2] have shown that despite being identified as parasites, viruses may have symbiotic relationships that are beneficial to the host as well. Viruses represent the most abundant biological entity in the biosphere with an estimated phage population of ~10^31^ [3]. Many microbiological experiments conducted in the past highlight the effect that viruses have on different processes in our biosphere. Examples include their effects on food web and organic carbon flow in the oceans [4], and population structure of bacterial communities in the human gut [5], [6]. The influence of viruses on driving ecological functionalities and evolutionary changes of prokaryotes has been previously highlighted, as well as the effect of viruses on the gene transfer across species [7]. One study [8] has illustrated the connection between the diversity of viruses and climate change with eight case studies, concluding that viruses are significantly influenced by climate change and in turn, are affecting biological processes contributing to climate changes. These studies stress the importance of studying viral ecology in different environments.

The conventional method of analysing the behaviour of viruses involves infecting them into cultured prokaryotic hosts. Such culture-dependent approaches are limited in applicability because a large number of microbial hosts have not been cultured [9]. One way of studying microbes in a culture-independent manner is the use of taxonomic marker genes like 16S ribosomal RNA gene (16S rRNA) that are conserved in genomes of all the species being studied [10]. However, due to the absence of such a conserved genomic region, the traditional marker genes based methods such as Polymerase Chain Reaction (PCR) and Fluorescence in situ hybridization (FISH) cannot be used to study viruses [9].

The emergence of Metagenomics helped in overcoming these challenges in studying the dynamics of viruses in different environments. Metagenomics refers to the biotechnological and bioinformatics methods involved in culture-independent analysis of genetic material of all microbial organisms in an environmental sample. A metagenome is the collection of genomic sequences of all the organisms in a given environment [9]. Advancements in high-throughput DNA sequencing and assembling techniques [11], [12], [13] have made metagenomics a popular approach for studying microbial ecology. The major steps involved in a metagenomics study have been previously reviewed [14] and include sample collection; extraction of DNA and removal of unwanted genetic material such as proteins, organelles and membranes; fragmentation of DNA using enzymes or mechanical techniques; sequencing of DNA; and bioinformatic analysis [14]. Metagenomics have a range of applications such as production of novel enzymes, discovery of new antibiotics and production of biosurfactants [15] and metagenomics related researches are being conducted around the world [16]. Moreover, metagenomics is expected to be highly effective in enteric disease diagnostics [17]. Bioinformatic analyses conducted on metagenomic data helps in expanding our knowledge on microbes in terms of taxonomic profiles, metabolic pathways and inter-species interactions etc. [18].

A metagenome of a viral population is termed a ‘metavirome’ [19]. The first metavirome study was an experiment carried out to study the ecology of viruses in marine environments using samples extracted from the two oceans Scripps Pier, CA and Mission Bay, San Diego. [20], [21]. Thereafter, many studies have been conducted to analyse metaviromes of samples collected from different environments such as sea water [20], [22], marine sediments [23], soil [24], human faeces [25], [26] and the human gut [27], [28], [29].

Biodiversity is an important ecological parameter in understanding the dynamics of a given environment as there is a strong relationship between biodiversity and the stability of an ecosystem [30]. It can be quantified in three ways: *α*-diversity referring to the diversity of a given sample or environment, *γ*-diversity quantifying the collective diversity of multiple environments and *β*-diversity capturing the difference in diversity among environments [31]. Implications of *α*, *β* and *γ* diversities have been reviewed comprehensively [32], [33]. One aspect often considered in a metagenomics study is *α*-diversity which is also termed ‘species diversity’.

The definition of a *virus species* has been debated [34], [35], and is being updated [36]. Generally, the term *species* is used to refer to the lowest category in biological classification; however, whether the term species should be referred to an individual entity or an abstract class or category remains debated [35]. Initially, the concept of species was considered to be not applicable for viruses because the early definition of species as *groups of interbreeding natural populations which are reproductively isolated from other such groups*, may not be related to viruses [34]. The International Committee on Taxonomy of Viruses (ICTV) which acts as the body responsible for maintaining the virus taxonomy [37], has accepted the formal definition of a virus species as “a polythetic class of viruses that constitutes a replicating lineage and occupies a particular ecological niche” [34], [38]. A *polythetic* class consists of members having multiple properties in common, but may not be defined by a single property [39]. Metagenomics can help in obtaining the assemblies of complete genome sequences of new viruses, however the obtained assemblies may lack information of their biological properties raising the concern how to define a virus species based on metagenomics data [36]. The term viral *genotype* has been used in the first metagenomic experiment of viruses [20] referring to in silico conditions, assuring that sequences of different phage genomes may not assemble together [20], [40]. The complexities in defining taxonomy of viruses as mentioned have been reviewed comprehensively [35] and implications of metagenomics in defining taxonomy of viruses is well documented [36]. In 2016, ICTV endorsed a proposal made to classify viruses solely based on metagenomics sequence data. This proposal recommends retaining the ICTV definition of a virus species and using biological characteristics that may be inferred from sequence data such as genome organization, replication strategy, presence of homologous genes and host range or type of vector [36].

Alternative approaches to quantify biodiversity instead of measures of species diversity have been proposed [41], [42]. An example is the suggestion to use statistical properties of communities with straightforward biological interpretations [41]. However, as far as metavirome studies are considered, estimation of species diversity is a key step in the bioinformatics analysis pipeline [43]. As far as viral communities are considered, species diversity indices may be used to answer a number of questions. Examples include: use of species diversity estimates to learn the relationship between species richness and range size distributions in plants [44], [45], demonstration of factors leading to the differences between the ambient and induced viral communities [46] considering species diversity of viruses, and prediction of zoonotic potential of mammalian viruses [47], modelling predator-prey dynamics based on rank-abundance distributions [48], use of evenness indices to determine factors affecting horizontal gene transfer and functional microbiome evolution in chicken cecum microbiome [49].

This review summarises the existing bioinformatics methods and tools for quantifying viral diversity from metavirome data. The widely considered species diversity measures in metavirome studies are defined and described in brief. The existing methods for estimating viral diversity measures are reviewed comparatively and their limitations are identified. Furthermore, possible alternative approaches are proposed to address the limitations in existing methods. Previous reviews have summarised various bioinformatics strategies used in existing methods for studying viruses [50], [51]. This review discusses further methods for measuring species diversity from metavirome data with comparisons between them.

## 2 Common Measures of Viral Diversity

Three commonly used species diversity measures in previous metavirome studies are species richness, Shannon-Wiener index and evenness. They represent the key quantitative species diversity measures: species richness, heterogeneity and equability [52]. The rank-abundance distribution and the relative abundances of genomes have also been considered (e.g.: [20], [53], [54], [55]).

Species richness is the total number of species in a population and is estimated from a sample, a representative subset of the population. While two environments may have equal species richness, if some species are dominant in number in one environment (i.e. less diverse) these two environments should be considered as different in diversity. Evenness captures how uniformly the species are distributed in number in an environment and is related with the relative abundances of species. If there are *n*_*i*_ number of individuals from *i*th species, its relative abundance, $f_{i}=n_{i}/\overset{M}{\underset{i=1}{\sum }}n_{i}$ where *M* is species richness. Heterogeneity measures combine species richness with evenness [52]. A commonly used heterogeneity measure is the *Shannon - Wiener* index. Shannon - Wiener index [56] considers both species richness and relative abundance and is defined as $H^{\prime }=-\overset{M}{\underset{i=1}{\sum }}f_{i}\;ln\;f_{i}$.

The equability indices are used to quantify the evenness of a community [52]. An example is Pielou's evenness. It is defined as *H*/*H*_*max*_, where *H* is a selected heterogeneity measure for the sample and *H*_*max*_ is the maximum possible value for *H*. For example, considering the Shannon - Wiener Index *H′*, evenness is calculated as *Evenness* = *H′*/*ln M* [57].

The underlying community structure is also frequently considered when studying diversity of an environment. The rank-abundance curve (also termed *Whittaker plot*) [58] is one way of visually representing the community structure based on the relative abundances of the species. On a rank-abundance plot, relative abundances of species are plotted against their abundance ranks. Abundance rank is determined by sorting the species based on their relative abundance and ranking them in decreasing order.

A set of methods has been proposed and implemented as tools to address the problem of estimation of viral diversity. An extensive review of statistical models and methods based on sampling theory for measuring the number of different classes (i.e. species richness) in a sample has been previously published [59]. This review suggests multiple approaches for estimating species richness including the use of parametric models, estimators of sample coverage and re-sampling methods etc. The models and methods developed based on those suggestions [60], [61], [62], [63] are being used to analyse bacterial populations [62], [64] and metaviromes [63] as discussed in the next section. However, due to the nature of fragment sampling methods employed in next generation sequencing when generating metaviromes, most of the mentioned suggestions cannot be readily used to analyse viral populations.

## 3 Methods for Measuring Species Diversity From Metavirome Data

**Fig. 1:**
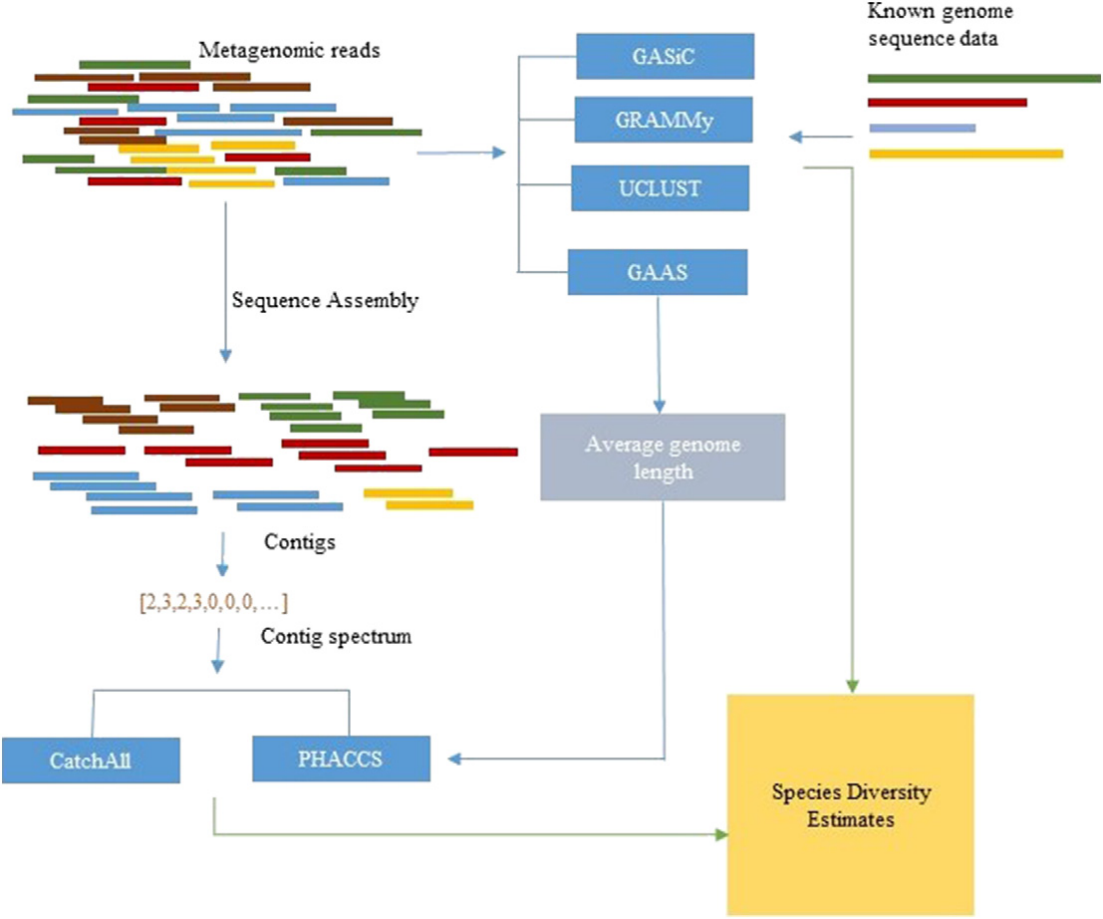
A schematic diagram summarising the stages where existing tools for measuring viral diversity can be integrated in a metagenomics data analysis pipeline.

Different strategies have been employed to measure viral diversity and assess their underlying community structure with the effective application of metagenomics in the study of viral populations. A summary of existing tools is given in Table 1 including species diversity measures that can be estimated using each tool. All these methods estimate species diversity measures from a given environmental sample using metagenomic sequences or assembled sequences (contigs) as the input (Fig. 1).

**Table 1:** Summary of existing tools for estimating species diversity measures in metavirome studies.

	Tool	Estimated species diversity measures	Published in	Resource
Sequence similarity- independent methods	PHACCS (20)	Species richness	2005	https://sourceforge.net/projects/phaccs/
		Shannon-Wiener index		
		Evenness		
		Rank-abundance distribution		
	CatchAll [63]	Species Richness	2012	http://www.northeastern.edu/catchall/
Sequence similarity-dependent methods	UCLUST [68]	Clusters of similar sequences	2010	http://www.drive5.com/usearch/
	GAAS [55]	Genome relative abundance	2009	https://sourceforge.net/projects/gaas/
	GRAMMy [69]	Genome relative abundance	2011	http://meta.usc.edu/softs/grammy/
	GASiC [70]	Genome relative abundance	2012	https://sourceforge.net/projects/gasic/

The existing techniques can be categorised into two as sequence similarity-dependent methods and sequence similarity-independent methods. The sequence data of viruses identified from previous metagenomics studies have been populated in public databases such as National Center for Biotechnology Information (NCBI) [65], viral RefSeq database (https://www.ncbi.nlm.nih.gov/genome/viruses) and METAVIR [66] server (http://metavir-meb.univ-bpclermont.fr/). The sequence similarity-dependent methods employ the data available in these reference databases. They estimate species diversity measures based on the results of a similarity comparison between sequences generated in the experiment and the sequences of already known genomes. In contrast, the sequence similarity-independent methods are based on statistical modelling of observed data and do not utilise comparisons with known sequences.

## 4 Sequence Similarity-independent Methods

The common strategy followed in sequence-similarity independent methods is to statistically model the observed data. The observed data that have been utilised in these methods is the ‘contig spectrum’. A set of overlapping genome sequences is termed a ‘contig’ and a contig spectrum is a vector where *n*_*i*_, the *i*th element denotes the number of contigs with *i* overlapping sequences. Contig spectrum can be determined from sequence assembly data that are available subsequent to shotgun cloning and DNA sequencing. Two tools have been implemented for measuring viral diversity in a sequence-similarity independent manner, namely PHACCS (PHAge Communities from Contig Spectrum) [67] and CatchAll [71]. A virus species is defined as a genotype in PHACCS [67] and a distinct group of viruses is considered as a species taxa in CatchAll [71].

A key strategy employing contig spectrum for viral diversity estimation is to firstly derive a model for the expected contig spectrum based on Lander-Waterman model for genome sequencing [72] considering different rank-abundance curve forms. Next, the model parameters, including the parameters of the assumed functional form of rank-abundance, giving the least error between expected and observed contig spectra can be estimated using maximum likelihood estimation and are used to calculate the species diversity measures. This strategy was first introduced in the metavirome study conducted to analyse the samples extracted from two oceans, Scripps Pier, CA and Mission Bay, San Diego [20] considering two rank-abundance curve forms (power-law and exponential) for estimations. Subsequently, a metavirome of a human faecal phage community has been analysed similarly assuming a power law distribution as the rank-abundance curve form [25]. When using this method, high stringency ought to be employed in sequence assembly conditions to ensure that a given contig occurs from the sequences belonging to the genomes of same phage or quite similar types [20]. Moreover, an assumed value is used for average genome length when deriving the model for expected contig spectrum. Later, this methodology has been implemented in the software, PHACCS [67].

PHACCS has been used in a number of metavirome studies [27], [67], [73], [74], [75] and may be considered as the state-of-the-art method. It can be used to estimate the species richness, evenness, Shannon-Weiner index and the parameters of the rank-abundance distribution. The user inputs required by PHACCS are the experimental contig spectrum, average genome length of the sample and a set of parameters related to sequencing and assembly (i.e. the number of DNA fragments being studied, the average DNA fragment size and the minimum overlap length considered in sequence assembly). An expression for the expected contig spectrum based on these input parameters is derived from a parametric model similar to [20]. PHACCS considers six rank-abundance curve forms in the computation and the best fitting parameters giving the least error between experimental and estimated contig spectra are calculated using maximum likelihood estimation. It provides a visualisation of community structure and the error details associated with the estimates. PHACCS may be regarded as the only tool facilitating estimation of all three aforementioned species diversity measures and visualisation of community structure of a metagenomic sample of viruses.

A method to estimate the species richness of a microbial community based on the frequencies of selected operational taxonomic units (OTU) s, named CatchAll [62] has been later adopted to estimate viral richness [63], [71]. In this approach, as the viruses lack a universal phylogenetic marker gene, frequencies of the contigs with a given number of overlapping sequences are used instead of the frequencies of OTUs [63], [71]. In the original approach of CatchAll, the observed frequency distribution of OTUs is first fitted into a set of parametric finite mixture models and coverage-based non-parametric models. Next, the species diversity measures are estimated from the best model (i.e. the model with the least error) from each (i.e. parametric and non-parametric models) and the overall best model. In addition to calculating the species richness estimates, CatchAll tool provides graphical representations of the corresponding parametric model, a performance comparison of different estimators considered and standard errors, confidence intervals, and goodness-of fit assessments associated with the estimates. Two differences between CatchAll and existing tools for calculating coverage-based nonparametric estimates have been identified [62]. Firstly, CatchAll can be used to determine the variation of estimates from coverage-based non-parametric model as more frequency counts are included in the data. Secondly it implements algorithms to compute standard errors and confidence interval values of the estimates with a higher accuracy than the other methods [62].

The use of CatchAll to estimate species richness of a metavirome using contig spectrum data has been proposed with CatchAll version 3.0 [63], [71]. The best overall estimate of species richness is given after computing twelve different estimates and assessing their errors [63]. The number of overlapping sequences observed *y*, is plotted against the number of overlapping sequences *x*, and the distribution is analysed to predict an estimate including the number of unobserved species, i.e. *y* value at *x* = 0. To improve the accuracy, a discounted estimate is calculated by adjusting the component with the highest frequency in the selected model.

A notable distinction between PHACCS and CatchAll is that PHACCS considers rank-abundance curve, while CatchAll considers the frequency count curve. Another distinction between PHACCS and CatchAll is that in parametric estimation of species richness, CatchAll considers the number of unobserved species which is calculated by curve fitting and projection. A comparison of richness estimates from CatchAll and PHACCS using 21 metaviromes from different environments demonstrate that estimates from CatchAll are consistent across the samples from similar environments and are higher than those from PHACCS [71]. Examples of applications of CatchAll to estimate viral richness are analysis of metaviromes from aquatic systems [76], [77] and rumen microbiome [78]. An evaluation using 100 simulated metaviromes has shown that PHACCS outperforms CatchAll in estimation accuracy [79]. This evaluation is discussed in more detail in Section 7.

## 5 Sequence Similarity-dependent Methods

Sequence similarity-dependent methods estimate the species diversity measures utilising sequence data available in reference databases. Firstly, the sequence reads generated in the experiment are compared against known genome sequences and their similarities are measured. Subsequently, measured similarity values are used to estimate species richness and relative abundances of known genomes within the sample. The tools, GAAS (Genome relative Abundance and Average Size) [55], GRAMMy (Genome Relative Abundance estimates based on Mixture Model theory) [69], GASiC (Genome Abundance Similarity Correction) [70] and UCLUST fall under this category (Table 1). All these methods separate sequences into clusters based on the nucleotide sequence similarity.

The BLAST algorithm is a widely used method for similarity searching between metagenomics reads and known genome sequences [80]. A parameter termed ‘E-value’ quantifies the significance of the similarity measures obtained by BLAST [81]. GAAS [55] tool introduces three steps to eliminate the limitations in conventional BLAST-based sequence comparisons and the biases that can be introduced in a BLAST search. First, only the sequences with a strong similarity to the reference sequences are considered based on maximum E-value, minimum similarity percentage and minimum relative alignment length. Second, the similarities are weighted based on the lengths of target genomes. Through normalisation based on the genome length, GAAS enables the consideration of single-stranded-RNA (ssRNA) viruses which are smaller, in the analysis. Thereby, GAAS improves the accuracy in estimating genetic diversity over a method based on conventional BLAST search [55]. The relative abundances of sequences in a metagenomic library is proportional to both the relative abundances and the genome lengths of the genomes in the sample [55]. Therefore, finally, the sum of weighted similarity of each genome is further normalised by its genome length to improve the accuracy of estimates. However, if a sequence read maps to multiple reference genomes, GAAS assigns it to a reference in an ad hoc manner. Consequently, the accuracy of estimates from GAAS is reduced in the dominant presence of such reads [69], [70]. GRAMMY [69] suggests mapping reads in a probabilistic manner, improving the accuracy of estimates of genome relative abundance. However, the similarities among the reference genomes could affect the accuracy of both GAAS and GRAMMY. GASiC [70] improves the estimation accuracy by correcting this bias. In GASiC, the initial abundances are estimated based on similarity to the reference genomes and then corrected based on similarities among the reference genomes. Quantification of viral RNA is challenging because many RNA viruses do not exist as a group of identical clones, but as groups of closely related variants (termed clouds of quasispecies [82]). The correction step based on similarities in reference genomes in GASiC has been demonstrated to be effective in quantifying viral RNA over considering only the reads similarity to reference genomes, without the correction step [70].

GAAS tool has been evaluated on 99 metaviromes with a similarity threshold less than that considered for bacterial, archeal and eukaryotic metagenomes [55]. Results from a simulation study have shown that the error in relative abundance estimates from GAAS increased from 0.0756 to 0.563 when the number of unknown species in the sample was increased from 0% to 80%. This finding highlights the importance of a comprehensive reference database.

Species richness can be estimated by identifying the number of similar groups after clustering reads based on their similarity to known genome sequences. Applicability of such strategies to estimate viral richness has been evaluated in [79] using UCLUST. UCLUST is a tool for fast sequence comparison and can be used to identify the number of similar groups based on read similarity [79], [83]. A faster sequence searching algorithm named USEARCH [83] is used in UCLUST to select matching clusters for a given sequence. The heuristic approach adopted in UCLUST identifies one or few better hits faster than finding all the homologous sequences and it has been shown to provide better results than BLAST [83]. The output from UCLUST is a set of clusters of similar sequences and is an indicator of the number of different species.

## 6 Software Implementations

Table 2 summarises implementation details of tools that have been discussed in this review. It lists the input data required by each tool and the programming language used. In addition, the operating system(s) that each tool supports and ways that they can be executed, either via Graphical User Interface (GUI) or Command Line Interface (CLI), are stated. All the tools are available as standalone software packages and hence support integration of them into a metagenomics analysis pipeline.

**Table 2:** Summary of implementation details of the existing tools.

Tool	Input data	Programmed in	Operating system/s supported	Interface
PHACCS [67]	Contig spectrum	Matlab, Perl	Linux,Mac OS, Windows	Web based GUI
	Average genome length			
	Sequencing and assembling settings			
CatchAll [63]	Contig spectrum	.Net Framework	Linux,Mac OS,Windows	GUI, CLI
UCLUST [68]	Metagenomic reads	–a	Linux, Mac OS, Windows	CLI
GAAS [55]	Metagenomic reads	Perl	Linux, Mac OS, Windows	CLI
GRAMMy [69]	Metagenomic reads	C++	Linux	CLI
		Python		
GASiC [70]	Metagenomics reads	Python	Linux	CLI

a Implementation details of the tool is not available.

Both PHACCS and CatchAll require a contig spectrum vector which can be computed after sequence assembly. In addition, PHACCS requires the sequencing and assembly parameters used in the experiment and a value for average genome length. If the latter is not provided, a value of 50 kbp is used by default. PHACCS may be executed from the command line and can also be deployed as a web-based tool with a GUI. CatchAll is a standalone package and can be executed either via the CLI or GUI. CatchAll may be considered more user-friendly than PHACCS as it requires only the contig spectrum vector.

UCLUST, GAAS, GRAMMy and GASiC require the metagenomics reads as the input. Since they implement sequence similarity-dependent strategies, they also require a database of reference genome sequences. They all provide execution from the command line only. Since GAAS can be used to estimate a value for average genome length, it can be integrated with PHACCS as a pre-step in estimating species diversity measures from PHACCS. A schematic diagram showing the steps where existing tools can be used in a metagenomics study is shown in Fig. 1.

## 7 Limitations of Existing Methods

Both sequence similarity-dependent and sequence similarity-independent methodologies discussed in this review pose limitations. Moreover, the applicability of a given approach may depend on the species diversity measures of interest. Use of contig spectrum employing a frequency count approach for estimating species richness as implemented in CatchAll has shown to result in richness estimates that are order of magnitude higher than the actual richness [79]. The accuracy of existing approach of statistical modelling of expected contig spectrum based on rank-abundance distribution forms is affected by its assumption on genome length distribution. Despite their limitations, approximations made using sequence-similarity dependent methods are useful in comparative studies of viral diversity in different environments [79]. Sequence similarity-dependent approaches are mainly limited by the amount of available reference genome sequence data. However, such approaches may effectively be used for inferring relative abundances of known viral types in a metavirome. These limitations of existing methodologies are discussed in detail in subsequent sections.

### 7.1 Unrealistic Estimates

We find the most recent evaluation of the accuracy of richness estimates from existing tools in [79] based on one set of simulated data and considering only PHACCS, CatchAll and UCLUST. Results from this study [79] indicate that estimated richness values from CatchAll and UCLUST are significantly higher than the estimates from PHACCS, which has resulted in the most accurate richness estimates. Normalisation of the estimates based on average genome length has improved the estimation accuracy of UCLUST; however, estimates from CatchAll have remained at least one order of magnitude higher than the expected value. When using contig spectrum to analyse a metavirome, CatchAll regards each contig as a viral type in contrast to the real world scenario where multiple contigs can be spawned from one genotype. This assumption could lead to erroneous higher richness estimates. An advantage of frequency count approach proposed with CatchAll is that it can be used to estimate the number of genotypes that are unobserved in the sample. However, its application to estimate species richness from metavirome data based on contig spectrum may lead to higher richness estimates.

### 7.2 Effect of Genome Length Distribution on Viral Diversity Estimates

When estimating viral diversity measures employing the model derived for expected contig spectrum based on Lander-Waterman model for genome sequencing to estimate viral diversity measures, an assumption is made on an average genome length. PHACCS implements a similar strategy and consequently requires a value for average genome length as user input. This assumption of all the genotypes in the sample are of the same size may affect the accuracy of estimated diversity measures due to two reasons: use of different methods to estimate an average genome length, the variation of genome lengths.

When using this method to estimate viral diversity measures, average genome length value has been determined in three ways in previous studies. One is to use an assumed value [84], [85] or the default value of 50 kbp [27], [67], [73], [86]. The use of 50 kbp as average genome length for marine viral populations is supported by previous research [87] but may not be applicable for viral populations from different environments. The estimates from PHACCS are sensitive to the average genome length and different assumptions can lead to different estimates [74], [84]. The second method of estimating average genome length is to use GAAS tool [55]. However, it is mainly limited by the amount of reference sequences available. The third method is to use the in vitro method of PFGE (Pulsed Field Gel Electrophoresis) [84], [87]. In PFGE, electrophoretic bands on an agarose gel are used to identify the spectrum of genome lengths. The estimated value from PFGE could be erroneous due to multiple genomes being represented in a single band [55].

The requirement of average genome length as user input has been identified as a limitation in PHACCS and has been addressed by a recently developed tool ENVirT [88]. ENVirT is based on a modelling approach similar to PHACCS but considers average genome length as a variable. A Genetic Algorithm based optimisation strategy is suggested in ENVirT to simultaneously estimate average genome length and species diversity measures by minimising the error between experimental and predicted contig spectra.

#### 7.2.1 Results From a Simulation to Determine the Effect of Variation of Genome Lengths on Accuracy of Estimates From PHACCS

**Fig. 2:**
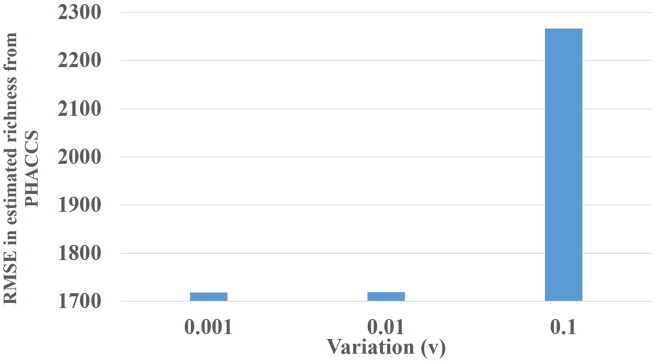
The effect of variation of genome lengths on the accuracy of species richness estimates from PHACCS.

Moreover, the variation of genome lengths of viruses in similar environments could be large. Therefore, assuming that all the genotypes in the sample are of identical size may affect the accuracy of estimated species diversity measures. In order to assess the effect of variation in genome lengths on estimates from PHACCS, contig spectra were simulated for 3 communities of *richness* = 10,000, mean genome length, *L* = 50 kbp and *evenness* = 0.81 having a power-law rank-abundance distribution and their genome lengths following a normal distribution *N*(*L*,(*Lv*)^2^) with varying *v*. The values of *v* considered are *v* = {0.001,0.001,0.1}. Ten contig spectra were generated from each community and Root Mean Squared Error (RMSE) of richness estimates from PHACCS are shown in Fig. 2. The results suggest that the variation of genome lengths of the population can significantly affect the accuracy of richness estimates that are calculated using the approach implemented in PHACCS.

### 7.3 Limited Mappings in Reference Databases

Sequence similarity-dependent approaches utilise data available in reference databases. However, as far as viruses are concerned, a larger proportion of the viruses in the environment is yet unknown. Consequently, in the absence of a comprehensive database of reference genomes, viral richness estimated by grouping sequences based on their similarity to already known genome sequences may be inaccurate.

However, sequence similarity-dependent approaches are useful in understanding the abundances of already known genomes in the environment under consideration. Such methods are also useful in time series experiments where the composition of the studied community is known from previous studies and a comprehensive set of reference sequences is available [70].

### 7.4 Analysis of RNA Viruses

The application of the methods discussed will be limited in analysing RNA viruses, mainly due to constraints of the experimental set-up. When isolating the viral community DNA, larger viruses and ssRNA viruses may be filtered out and their sequences may not be included in the contig spectrum [20], [67]. Consequently, ssRNA will be omitted when estimating species richness based on the contig spectrum [67]. The tools GAAS and GASiC have steps implemented to effectively analyse RNA viruses (Section 5), however their estimates on RNA data may be lower than their estimates based on DNA data [70].

### 7.5 Effect of Microdiversity

Microdiversity refers to the diversity of closely related organisms [89]. Recent research suggest that microdiversity affects the metagenomic sequence assembly and more reads remain unassembled as the microdiversity of a sample is increased [90]. Similarity-independent methods that are based on the contig spectrum considers the unassembled reads when calculating the diversity estimates. However, they do not include a correction for the contigs belonging to the same virus being placed into separate contigs due to limitations in the assembly. Consequently, similarity-independent methods may provide higher estimates than the real diversities in the presence of microdiversity.

## 8 Summary and Outlook

Viruses play an integral part in the ecology of different environments and metavirome studies have enabled the effective study of viruses associated with these environments in a culture-independent manner. Frequently considered viral diversity measures in metavirome studies include species richness, Shannon-Wiener index, evenness and rank-abundance distribution. Existing methods for estimating species diversity measures from metavirome data may be categorised into two as sequence similarity-dependent methods and sequence similarity-independent methods. Sequence similarity-dependent methods are based on similarity measures calculated by comparing the sequences generated in the experiments against the sequence data available in reference databases. In contrast, species diversity estimates calculated employing sequence similarity-independent methods do not depend on read similarity to known genome sequences.

Sequence similarity-dependent methods are useful in identifying the abundances of known genomes in a metavirome. Improving the accuracy of these methods will help to evaluate the diversity of known genotypes in a given environment. However, their application for analysing viruses in a given environment, may be limited by the amount of reference sequence data available. Therefore, the availability of reference databases and their continuous update is crucial in making sequence similarity-dependent approaches applicable for viral diversity estimation.

Sequence similarity-independent approaches mainly use contig spectrum to estimate species diversity measures. However, existing frequency count approach has shown to result in richness estimates higher than the expected values. The approach employing rank-abundance distribution forms is limited by its requirement of an average genome length of the sample which is not readily available. Its accuracy is also affected by the variation in genome length of the sample. Development of alternative models based on additional data that are readily available (such as sequencing depth) and suitable optimisation strategies will alleviate the limitations associated with sequence similarity-independent approaches.

Recent metavirome studies have investigated protein-clustering to identify groups of similar species [91], [92], [93]. In protein-clustering, the assembled reads are clustered based on their corresponding protein similarities. The methodology UCLUST which enables clustering of nucleic acid sequences can also be used to generate protein-clusters [93]. Estimation of functional diversity of a metavirome and its comparison between metaviromes of other environments can be effectively performed based on protein-clusters [93]. Therefore, coupling species diversity measures with protein-cluster analysis of metaviromes would broaden the knowledge on ecology of viruses in different environments.

The microdiversity of a metavirome affects the metagenomic sequence assembly. Future work on its effect on accuracy of species diversity methods using current methods will be beneficial in development of robust methods to analyse environments with (low to high) varying levels of microdiversity.

A broader knowledge of viral diversity in a given environment may be obtained by considering estimates from both similarity-dependent methods and similarity-independent methods [53], [94]. A study has analysed 31 metaviromes from different environments (hypersaline, marine, freshwater and eukaryote) considering estimates from both PHACCS and UCLUST. The mentioned study has shown that the environments are similar in number of virotypes, but differ in genetic diversity (number of clusters of similar genes) [53]. Another study on human skin virome has considered species diversity estimates from both PHACCS and GAAS. The estimates from GAAS have been lower than estimates from PHACCS. Moreover, estimates from GAAS have been similar across the considered environments whereas PHACCS estimates have been different in different environments. The observed differences between estimates from GAAS and PHACCS may be due to the limited availability of reference sequences for GAAS. A previous study has evaluated the accuracy of species richness estimates from existing methods using simulated data. However, it considers only three implementations of existing methods. A comprehensive analysis of available methods in terms of their performance based on real data and accuracy based on simulated data will provide a better understanding for a user to choose between these methods and use them in a complementary manner.

## Conflicts of Interest

None.
